# The natural history of *de novo* donor-specific HLA antibodies after kidney transplantation

**DOI:** 10.3389/fmed.2022.943502

**Published:** 2022-09-16

**Authors:** Covadonga López del Moral, Kaiyin Wu, Marcel Naik, Bilgin Osmanodja, Aylin Akifova, Nils Lachmann, Diana Stauch, Sabine Hergovits, Mira Choi, Friederike Bachmann, Fabian Halleck, Eva Schrezenmeier, Danilo Schmidt, Klemens Budde

**Affiliations:** ^1^Department of Nephrology and Medical Intensive Care, Charité – Universitätsmedizin Berlin, Corporate Member of Freie Universität Berlin and Humboldt-Universität zu Berlin, Berlin, Germany; ^2^Valdecilla Biomedical Research Institute (IDIVAL), Santander, Spain; ^3^Department of Pathology, Charité – Universitätsmedizin Berlin, Berlin, Germany; ^4^Institute for Transfusion Medicine, HLA-Laboratory, Charité – Universitätsmedizin Berlin, Berlin, Germany; ^5^Berlin Institute of Health Charité – Universitätsmedizin Berlin, BIH Academy, Berlin, Germany

**Keywords:** donor-specific antibodies, mean fluorescence intensity, graft failure, antibody-mediated rejection, kidney transplantation

## Abstract

**Background:**

*De novo* donor-specific HLA antibodies (dnDSA) are key factors in the diagnosis of antibody-mediated rejection (ABMR) and related to graft loss.

**Methods:**

This retrospective study was designed to evaluate the natural course of dnDSA in graft function and kidney allograft survival and to assess the impact of mean fluorescence intensity (MFI) evolution as detected by annual Luminex^®^ screening. All 400 kidney transplant recipients with 731 dnDSA against the last graft (01/03/2000-31/05/2021) were included.

**Results:**

During 8.3 years of follow-up, ABMR occurred in 24.8% and graft loss in 33.3% of the cases, especially in patients with class I and II dnDSA, and those with multiple dnDSA. We observed frequent changes in MFI with 5-year allograft survivals post-dnDSA of 74.0% in patients with MFI reduction ≥ 50%, 62.4% with fluctuating MFI (MFI reduction ≥ 50% and doubling), and 52.7% with doubling MFI (log-rank *p* < 0.001). Interestingly, dnDSA in 168 (24.3%) cases became negative at some point during follow-up, and 38/400 (9.5%) patients became stable negative, which was associated with better graft survival. Multivariable analysis revealed the importance of MFI evolution and rejection, while class and number of dnDSA were not contributors in this model.

**Conclusion:**

In summary, we provide an in-depth analysis of the natural course of dnDSA after kidney transplantation, first evidence for the impact of MFI evolution on graft outcomes, and describe a relevant number of patients with a stable disappearance of dnDSA, related to better allograft survival.

## Introduction

Short-term graft survival has improved over the past decades in kidney transplantation, but no major changes in long-term survival have been achieved (1–4). Antibody-mediated rejection (ABMR) is an important cause of graft failure (5–11). Although non-HLA antibodies may also cause graft dysfunction (12–15), it is well-known that preformed or *de novo* HLA donor-specific antibodies (dnDSA) are strongly associated with rejection and graft failure (16–22). The development of *dn*DSA may occur at any time after transplantation, and different characteristics of DSA may determine the clinical phenotype of rejection (23–29). The presence of dnDSA has been reported in 13–27% of previously non-sensitized patients, but the indication and frequency of systematic DSA screening in stable patients are not currently established (30–32). High HLA mismatch is one of the risk factors for dnDSA development (33–36). Non-adherence to treatment, under-immunosuppression, and graft inflammation are other factors that are related to dnDSA formation (29). It has been reported that the presence of both class I and II dnDSAs is more strongly related to graft failure, but few studies have specifically analyzed the long-term effects of antibody class (27, 37–43), and the impact of the number of dnDSA per patient on graft survival is unknown.

The Luminex^®^-based single-antigen bead (SAB) assay is currently the most appropriate method for the detection of HLA antibodies, which allows for semiquantitative analysis of the level of anti-HLA antibodies by the mean fluorescence intensity (MFI) (44–46). It is assumed that antibodies with higher MFI values are more harmful and related to graft dysfunction, but the relationship between clinical outcomes and MFI level is not fully established. The correlation between MFI and the amount of bound HLA antibodies is not linear and can be affected by several factors, such as the inhibitory effect produced by complement (prozone effect) (45, 47, 48). Currently, there is no accepted MFI value that is clinically significant, and each laboratory has set its own MFI positivity threshold (32, 41, 46). The STAR 2017 Working Group (32) gave recommendations for HLA antibody testing, pointing out that differences of up to 25% or even 50% in MFI values should not be considered meaningful.

The purpose of the current study was to evaluate the natural history and clinical evolution of patients with dnDSA after kidney transplantation. We wanted to specifically address the relationship of dnDSA MFI values with graft failure. Changes in renal function were evaluated to assess the evolution of these analytical parameters after the occurrence of dnDSA.

## Materials and methods

### Patient population

For this retrospective analysis, we included all kidney transplant recipients with dnDSA from 01/03/2000 until 31/05/2021 (end of follow-up) at Charité-Universitätsmedizin Berlin (Germany). All patients with dnDSA against the last graft with complete HLA typing were included, excluding those patients with preformed DSA before transplantation. The primary outcome variable in our study was time to death-censored graft failure, defined as graft loss (i.e., the need for permanent dialysis, allograft nephrectomy, or re-transplantation). Patients who developed dnDSA after graft loss were excluded.

All data including estimated glomerular filtration rate (GFR, ml/min), proteinuria (mg/g creatinine), delayed graft function (DGF), defined as the need for dialysis within 7 days of transplant, and biopsy data were collected from the prospectively maintained database (TBase) (49). All rejections were categorized according to Banff 2017 classification (5, 50, 51). Calculated panel-reactive antibody (cPRA) was obtained through the Virtual PRA Calculator of the Eurotransplant Reference Laboratory.^1^ No institutional review board approval was required for this retrospective analysis.

### *De novo* donor-specific HLA antibodies

Regular annual monitoring of HLA antibodies was performed as described previously (26, 33) and in case of clinical signs of impaired allograft function. DnDSA were determined by Luminex^®^ -based LABScreen^®^ SAB assay (One Lambda, Canoga Park, CA). The general MFI positivity threshold in our laboratory was 1,000. Despite this, the first occurrence date in our study was defined as the date of the medical report by the immunology department in which dnDSA was first assigned, considering other factors such as plausibility (52) and evolution of HLA antibodies posttransplant, regardless of MFI value. The most probable two-field HLA typing of the donor (53) was considered to assign DSA and the respective MFI as appropriate as possible. For missing information on specific HLA loci (usually DQA and DPA), DRB1∼DQA1∼DQB1 and DPA1∼DPB1 haplotype frequencies were used to assign the most probable allele, according to extended haplotype frequencies previously described in the European population (54–56). The first appearance of each dnDSA and the date of the last negative sample were collected. Because each dnDSA had its own time of the first occurrence and its own MFI evolution, we also performed some analyses for different dnDSA as indicated.

*De novo* DSAs were categorized according to MFI on the date of the first occurrence (<500, 500–999, 1,000–2,999, 3,000–9,999, and ≥10,000), and they were also classified according to MFI evolution in the subsequent samples [MFI increase ≥ 50%, MFI reduction ≥ 50%, fluctuating MFI (increase and reduction ≥ 50%)]. In dnDSA with ≥ 50% MFI reduction. specific active treatment for ABMR was recorded (57), excluding changes in chronic baseline immunosuppression. The frequency of negativity (MFI < 500) after the first occurrence of each dnDSA was analyzed, either temporary or stable negativity.

### Statistical analysis

Continuous variables were expressed as mean ± standard deviation (SD) or median and interquartile range (IQR) according to their distribution. Categorical variables were described as relative frequencies. A non-parametric test (Mann–Whitney *U* test) was used to compare variables with non-normal distribution. A chi-square test was used to compare the average values of categorical variables. Univariable and multivariable Cox regression analyses were performed to determine which clinical variables were associated with death-censored graft loss, and hazard ratios (HR) were reported with 95% confidence intervals. Missing laboratory values due to graft loss or lack of follow-up after dnDSA appearance were imputed using last observation carried forward (LOCF) analysis and automatic multiple imputation (MI) using five default imputations. Time-to-event outcome data were assessed by Kaplan–Meier plots and log-rank tests. *P* < 5% defined statistical significance. Statistical analysis was conducted using the SPSS statistical software package (IBM SPSS Statistics, Version 25.0. Armonk, NY: IBM Corp.).

## Results

**FIGURE 1:**
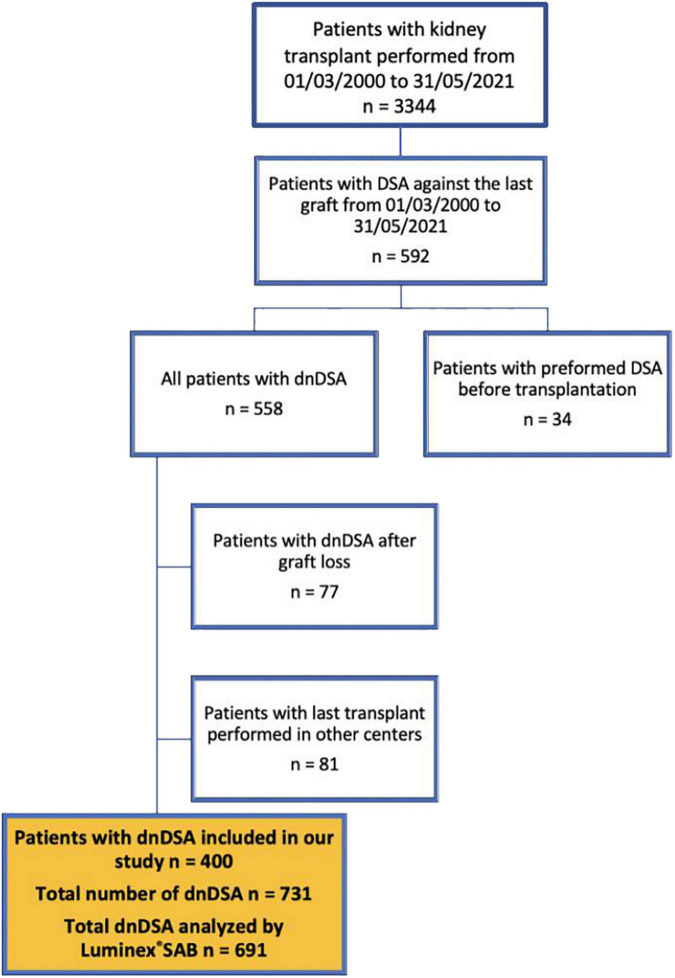
Flowchart of the patients in our study. DSA, donor-specific antibodies; dnDSA, *de novo* donor-specific antibodies; SAB, single antigen bead.

In total, we identified 400 patients with dnDSA (Figure 1), which accounts for 11.9% of the total population of 3,344 transplanted patients in the period from March 2000 until May 2021. The study cohort comprised mainly patients with a first single-kidney transplant from a deceased donor (Table 1) with a median follow-up of 8.3 years (IQR 5.5–10.7) after dnDSA appearance. By design of the study, none of the patients had DSA at the time of transplantation, and only a few were sensitized. Patients with dnDSA in our study had significantly lower long-term allograft survival compared to patients without dnDSA (Control group, *n* = 2,752), as shown in Supplementary Figure 1.

**TABLE 1:** Baseline characteristics of patients with dnDSA.

Variables	Patients with dnDSA (*n* = 400)
Recipient age at time of TX	46.1 (34.2–59.1)
Recipient sex (male, %)	62.5% (*n* = 250)
Follow-up (years) after TX	12.9 (9.6–16.3)
Follow-up (years) after dnDSA development*	8.3 (5.5–10.7)
Graft loss (%)	33.3% (*n* = 133)
• Time (years) from TX to graft loss	• 8.4 ± 4.9
• Time (years) from dnDSA to graft loss*	• 4.6 (1.7–8.1)
Death (%)	24.0% (n = 96)
• Time (years) from TX to death	• 8.9 ± 4.3
Patients alive with functioning graft (%)	53.0% (*n* = 212)
Donor age	50.0 (39.0–59.5)
Donor sex (male,%)	51.0% (*n* = 204)
Donor blood type	
• A	• 39.4% (*n* = 158)
• B	• 13.3% (*n* = 53)
• AB	• 5.8% (*n* = 23)
• 0	• 41.5% (*n* = 166)
Donor type	
• Deceased donor (100% DBD)	• 68.5% (*n* = 274)
• Living donor	• 31.5% (*n* = 126)
First kidney transplant (%)	88.7% (*n* = 355)
Combined transplant (%)	6.8% (*n* = 27)
	• 5.5% (*n* = 22): Pancreas-kidney transplant
	• 1.3% (*n* = 5): Liver-kidney transplant
Cold ischemia time (CIT, minutes)	420.0 (165.0–768.0)
Delayed graft function (DGF, %)	29.7% (*n* = 119)
•cPRA ≥ 5% at the time of TX (%) (Eurotransplant)	16.5% (*n* = 66)
•cPRA ≥ 85% at the time of TX (%) (Eurotransplant)	5.8% (*n* = 23)
cPRA ≥ 5% at the time of TX (%)	
•cPRA ≥ 5% class I (%)	• 16.8% (*n* = 67)
•cPRA ≥ 5% class II (%)	• 11.3% (*n* = 45)
cPRA ≥ 85% at the time of TX (%)	
•cPRA ≥ 85% class I (%)	• 3.8% (*n* = 15)
cPRA ≥ 85% class II (%)	• 2.5% (*n* = 10)
Initial IS	
•Triple standard therapy (calcineurin inhibitor, mycophenolate, and steroids)	• 24.5% (*n* = 98)
•Triple standard therapy + anti-IL2R	• 49.8% (*n* = 199)
•Triple standard therapy + ATG	• 5.8% (*n* = 23)
Others	• 19.9% (*n* = 80)
HLA mismatch A = 0 (%)	30.5% (*n* = 122)
HLA mismatch A = 1 (%)	51.9% (*n* = 208)
HLA mismatch A = 2 (%)	17.6% (*n* = 70)
HLA mismatch B = 0 (%)	12.2% (*n* = 49)
HLA mismatch B = 1 (%)	50.9% (*n* = 203)
HLA mismatch B = 2 (%)	36.9% (*n* = 148)
HLA mismatch DRB1 = 0 (%)	10.7% (*n* = 43)
HLA mismatch DRB1 = 1 (%)	60.3% (*n* = 241)
HLA mismatch DRB1 = 2 (%)	29.0% (*n* = 116)
HLA mismatch DQB1 = 0 (%)	11.0% (*n* = 44)
HLA mismatch DQB1 = 1 (%)	57.8% (*n* = 231)
HLA mismatch DQB1 = 2 (%)	31.2% (*n* = 125)
Graft nephrectomy (%) after dnDSA occurrence	10.3% (*n* = 41)
• Cause of graft nephrectomy	
° Acute rejection	° 14.6% (*n* = 6)
° Chronic rejection	° 56.1% (*n* = 23)
° Surgical complications	° 4.9% (*n* = 2)
° Others	° 24.4% (*n* = 10)
• Time (months) from TX to graft nephrectomy	° 77.3 (30.7–138.1)
Patients with allograft kidney biopsy (%) (all by clinical indication; independent of results)	72.0% (*n* = 288)
•Patients with allograft kidney biopsy after dnDSA occurrence*	• 63.9% (*n* = 184)
Number of allograft kidney biopsy per patient	1.0 (0.0–3.0)
Number of dnDSA per patient	1.0 (1.0–2.0)
Patients with ≥ 2 dnDSA (independent of class) (%)	43.5% (*n* = 174)
Patients with ≥ 4 dnDSA (independent of class) (%)	10.3% (*n* = 41)
Class dnDSA per patient	
• Patients with class I dnDSA only (%)	• 18.5% (*n* = 74)
• Patients with class II dnDSA only (%)	• 59.3% (*n* = 237)
• Patients with both class I and II dnDSA (%)	• 22.3% (*n* = 89)
Proteinuria (mg/g creatinine) at the time of first occurrence of dnDSA*	182.0 (100.2–502.0)
Patients with proteinuria ≥500 mg/g creatinine at the time of first occurrence of dnDSA (%)*	21.3% (*n* = 85)
eGFR (ml/min) at the time of first occurrence of dnDSA*	41.0 (29.0–54.2)
Creatinine (mg/dl) at the time of first occurrence of dnDSA*	1.6 (1.3–2.3)
TCMR before first occurrence of dnDSA (%)*	35.0% (*n* = 140)
TCMR (all episodes, independent of first occurrence of dnDSA) (Banff 2017 Classification)	45.8% (*n* = 183)
•Acute TCMR borderline	• 27.3% (*n* = 50)
• Acute TCMR IA	• 13.1% (*n* = 24)
• Acute TCMR IB	•8.2% (*n* = 15)
•Acute TCMR IIA	• 12.0% (*n* = 22)
•Acute TCMR IIB	• 2.2% (*n* = 4)
• Acute TCMR III	• 0.5% (*n* = 1)
•Episodes of different categories per patient	•36.7% (*n* = 67)
ABMR (all episodes, independent of first occurrence of dnDSA) (Banff 2017 Classification)**	24.8% (*n* = 99)
• Active ABMR	• 16.2% (*n* = 16)
• Chronic active ABMR	• 59.6% (*n* = 59)
• Chronic ABMR	• 10.1% (*n* = 10)
• Episodes of different categories per patient	• 14.1% (*n* = 14)

Variables with normal distribution: mean ± SD. Variables with non-normal distribution: median and IQR. *At the time of occurrence of the first dnDSA for patients with > 1 dnDSA.

**All episodes of ABMR appeared at the time and/or after dnDSA the first occurrence. TX, transplant; dnDSA, *de novo* donor-specific antibody; DBD, donation after brain death; cPRA, calculated panel-reactive antibody; IS, immunosuppression; Anti-IL2R, anti-interleukin-2 receptor; ATG, antithymocyte globulin; HLA, human leukocyte antigen; eGFR, estimated glomerular filtration rate; TCMR, T-cell-mediated rejection; ABMR, antibody-mediated rejection.

Regular annual DSA screening was performed for more than 18 years (26), with a median number of 1.6 (IQR 1.2–2.0) DSA determinations per patient/year. The median time from the last negative sample to the first positive dnDSA was 11.3 (IQR 4.7–20.3) months.

The median number of dnDSA per patient was 1.0, but 10.3% of patients had ≥4 dnDSA. In patients with >1 dnDSA (*n* = 174, 43.5%), 113 (64.9%) had all dnDSA with the same date of appearance. In the other patients (*n* = 61, 35.1%), the median time from the first occurrence to the next first appearance of other dnDSA was 14.4 months (IQR 4.7–43.7) (Supplementary Figures 2–4).

**FIGURE 2:**
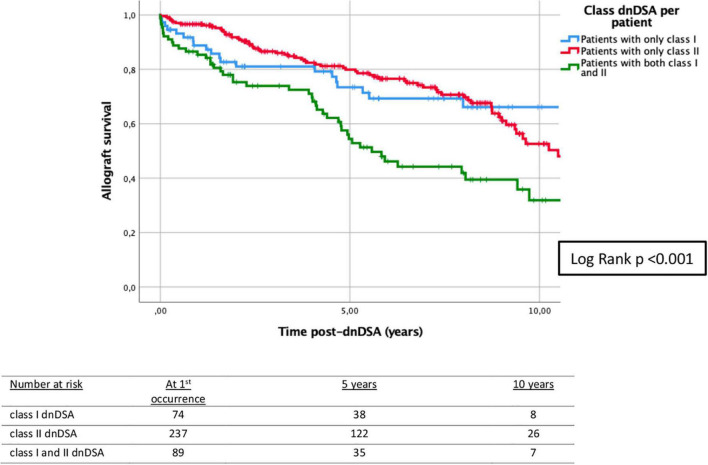
Kaplan–Meier survival analysis of death-censored graft failure for HLA class of dnDSA after the first occurrence of the first dnDSA. Five-year death-censored allograft survival post-dnDSA: 73.4% (±5.6%) for patients with class I dnDSA; 79.9% (±2.9%) for patients with class II dnDSA; and 54.4% (±5.9%) for patients with both class I and II dnDSA. Log-rank test *p* < 0.001. dnDSA, *de novo* donor-specific antibodies.

**FIGURE 3:**
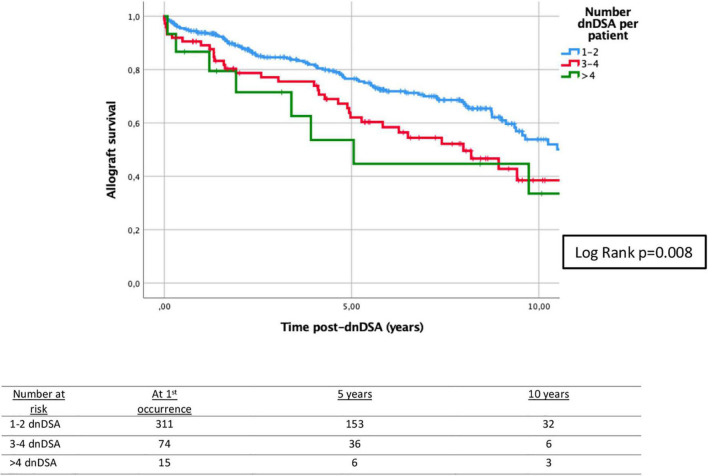
Kaplan–Meier survival analysis of death-censored graft failure for the number of dnDSA/patient after the first occurrence of the first dnDSA. Five-year death-censored allograft survival post-dnDSA: 76.6% (±2.7%) for patients with 1–2 dnDSA; 62.1% (±6.1%) for patients with 3–4 dnDSA; and 53.6% (±14.2%) for patients with > 4 dnDSA. Log-rank *p* = 0.008. dnDSA, *de novo* donor-specific antibodies.

The biopsies of allograft kidneys were performed by clinical indication (rise in creatinine and/or proteinuria), and 72.0% of patients had at least one biopsy (Table 1). About 35.0% of patients had at least one episode of T-cell mediated rejection (TCMR) before the first appearance of dnDSA. All episodes of ABMR appeared at the time and/or after the first occurrence of dnDSA (Supplementary Figure 5). Only 26/400 (6.5%) patients had rejection at the time of the first appearance of dnDSA, which, however, accounted for 24.8% of all ABMR episodes. Patients with at least one rejection episode, either TCMR or ABMR, had significantly lower graft survival compared to those patients without rejection, as shown in Supplementary Figures 6, 7. Analyzing the class of dnDSA, 18.5% of the patients presented only class I, 59.3% presented only class II, and 22.3% had both class I and II dnDSA. In patients with DQ-dnDSA (*n* = 260), 64.2% (*n* = 167) had only DQ-dnDSA, and 35.8% (*n* = 93) had DQ along with other dnDSA. In the latter group, most of the patients presented DQ at the time or before the appearance of other dnDSA (*n* = 79, 84.9%). These 79 patients had additional class I (50.6%), class II (27.8%), and both class I and II (21.5%) dnDSA. In patients with DQ-dnDSA which appeared before other dnDSA (*n* = 19), the median time from DQ to the occurrence of other dnDSA was 15.1 months (IQR 6.9–20.0). The class of dnDSA was associated with 5-year death-censored allograft survival (Figure 2). Similarly, death-censored allograft survival was related to the number of dnDSA (Figure 3).

Stratification by dnDSA (*n* = 731) (Table 2) revealed 231 (31.6%) class I and 500 (68.4%) class II dnDSA, including 363 class II-DQ dnDSA (72.6% of class II dnDSA). The median time from transplantation to the first occurrence of each dnDSA was 35.9 months, without significant differences between class I and II (*p* = 0.575) (Supplementary Figure 8).

**TABLE 2:** Characteristics of dnDSA.

Variables	All dnDSA (*n* = 731)	dnDSA class I (*n* = 231)	dnDSA class II (*n* = 500)	*p*
HLA mismatch A:				**0.001**
HLA mismatch A = 0 (%)	28.8%	19.7%	33.0%	
HLA mismatch A = 1 (%)	52.4%	60.7%	48.6%	
HLA mismatch A = 2 (%)	18.8%	19.7%	18.4%	
HLA mismatch B:				**0.002**
HLA mismatch B = 0 (%)	10.1%	6.1%	11.9%	
HLA mismatch B = 1 (%)	50.1%	45.9%	52.0%	
HLA mismatch B = 2 (%)	39.8%	48.0%	36.0%	
HLA mismatch DRB1:				**<0.001**
HLA mismatch DR = 0 (%)	8.4%	15.7%	5.1%	
HLA mismatch DR = 1 (%)	60.4%	56.8%	62.1%	
HLA mismatch DR = 2 (%)	31.1%	27.5%	32.8%	
HLA mismatch DQB1:				**0.002**
HLA mismatch DQ = 0 (%)	9.2%	14.1%	6.9%	
HLA mismatch DQ = 1 (%)	56.9%	58.1%	56.3%	
HLA mismatch DQ = 2 (%)	33.9%	27.8%	36.8%	
Time (months) from TX to first occurrence of dnDSA	35.9 (14.2–84.7)	35.0 (12.8–85.2)	38.1 (14.2–84.7)	0.575
Time (months) from last negative sample to sample with positive dnDSA	11.3 (4.7–20.3)	9.2 (3.2–19.5)	11.5 (5.6–22.4)	0.120
ABMR (at the time or after each dnDSA) (Banff 2017 Classification)	29.1%	29.4%	29.0%	0.904
Categories: • Active ABMR	• 14.6%	• 16.2%	• 13.8%	**<0.001**
• Chronic active ABMR	• 48.4%	• 33.8%	• 55.2%	
• Chronic ABMR	• 6.1%	• 1.5%	• 8.3%	
• Episodes of different previous categories	• 30.9%	• 48.5%	• 22.7%	

Variables with non-normal distribution: median and IQR. HLA, human leukocyte antigen; TX, transplant; dnDSA, *de novo* donor-specific antibody; ABMR, antibody-mediated rejection.

Analyzing MFI at the time and after the first occurrence in Luminex-defined dnDSA (*n* = 691; Table 3), we had 6.0 (IQR 4.0–9.0) samples/dnDSA with a median time between samples of 9.0 months (IQR 5.8–11.5). About 24.0% of dnDSA had doubling MFI during follow-up, in 36.9% we observed ≥50% MFI reduction, and 7.5% of dnDSA had fluctuating MFI. Analyzing these results per patient, 27.5% of patients had at least one dnDSA with doubling MFI, 42.5% with ≥50% MFI reduction, and 10.3% with fluctuating MFI. In dnDSA with ≥ 50% MFI reduction (*n* = 255), 25.5% (*n* = 65) had received some form of treatment (26), but 74.5% (*n* = 190) had a ‘spontaneous’ reduction. Interestingly, 168 (24.3%) dnDSA became negative at some point during follow-up and 100 (14.5%) dnDSA became stable negative. Altogether, 38/400 (9.5%) patients became stable negative.

**TABLE 3:** MFI values at the first occurrence and MFI evolution of dnDSA analyzed by Luminex^®^.

Variables	All dnDSA (*n* = 691)	dnDSA class I (*n* = 221)	dnDSA class II (*n* = 470)	*p*
MFI at first occurrence of dnDSA				**<0.001**
• 1: MFI < 500	• 2.5%	• 5.0%	• 1.3%	
• 2: MFI 500–999	• 11.1%	• 21.7%	• 6.2%	
• 3: 1,000–2,999	• 30.5%	• 41.6%	• 25.3%	
• 4: 3,000–9,999	• 36.5%	• 28.1%	• 40.4%	
• 5: >10,000	• 19.4%	• 3.6%	• 26.8%	
MFI evolution of dnDSA after first occurrence^∧^				0.080
• 1: MFI doubling	• 24.0%	• 23.1%	• 24.5%	
• 2: MFI reduction ≥50%	• 36.9%	• 41.2%	• 34.9%	
° Specific active treatment for ABMR*	° 25.5% (*n* = 65)	° 26.4% (*n* = 24)	° 25.0% (*n* = 41)	
• 3: MFI fluctuating (MFI doubling and reduction ≥50% at some point)	• 7.5%	• 9.5%	• 6.6%	
• 4: Other	• 24.0%	• 18.1%	• 26.8%	
• 5: No MFI evolution available	• 7.5%	• 8.1%	• 7.2%	
dnDSA becomes negative (MFI < 500) at some point during evolution	24.3%	37.1%	18.3%	**<0.001**
dnDSA becomes constant negative (MFI < 500) (Stable negative)**	14.5%	23.1%	10.4%	**<0.001**

^∧^MFI evolution independent of biopsy-proven rejection and treatments. *p-value = 0.809. **Stable negative dnDSA defined as MFI < 500 in every sample after the first negative sample. MFI, mean fluorescence intensity; dnDSA, *de novo* donor-specific antibody; ABMR, antibody-mediated rejection.

The relationship between MFI evolution and graft loss is shown in Table 4. The number of dnDSA with doubling and fluctuating MFI was higher in the graft loss group (*p* < 0.001), and temporary and stable MFI negativity was significantly lower in the graft loss group (*p* = 0.034 and 0.004).

**TABLE 4:** MFI values at the first occurrence and MFI evolution of dnDSA analyzed by Luminex^®^ and relationship with graft loss.

Variables	All dnDSA *n* = 691	No graft loss (*n* = 430)	Graft loss (*n* = 261)	*p*
MFI at first occurrence of dnDSA				0.563
• 1: MFI < 500	• 2.5%	• 2.3%	• 2.7%	
• 2: MFI 500–999	• 11.1%	• 10.7%	• 11.9%	
• 3: 1,000–2,999	• 30.5%	• 32.8%	• 26.8%	
• 4: 3,000–9,999	• 36.5%	• 34.9%	• 39.1%	
• 5: >10,000	• 19.4%	• 19.3%	• 19.5%	
MFI evolution of dnDSA after first occurrence^∧^				**<0.001**
• 1: MFI doubling	• 24.0%	15.1%	38.1%	
• 2: MFI reduction ≥ 50%	• 36.9%	44.4%	24.5%	
• 3: MFI fluctuating (MFI doubling and reduction ≥ 50% at some point)	• 7.5%	4.9%	11.9%	
• 4: Other	• 24.0%	25.6%	21.5%	
• 5: No MFI evolution available	7.5%	10.0%	3.4%	
dnDSA becomes negative MFI < 500 at some point during evolution	24.3%	27.2%	19.8%	**0.034**
dnDSA becomes constant negative (MFI < 500) (Stable negative)*	14.5%	17.4%	9.6%	**0.004**

^∧^MFI evolution independent of biopsy-proven rejection and treatments. *Stable negative dnDSA defined as MFI < 500 in every sample after the first negative sample. MFI, mean fluorescence intensity; dnDSA, *de novo* donor-specific antibody.

**FIGURE 4:**
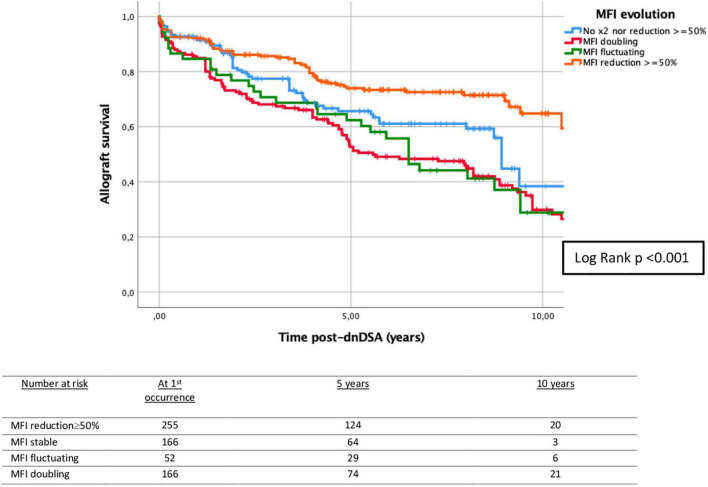
Kaplan–Meier survival analysis of death-censored graft failure for dnDSA-MFI evolution after the first occurrence of dnDSA. Five-year death-censored allograft survival post-dnDSA: 74.0% (±3.0%) when MFI reduction ≥ 50%; 65.6% (±4.2%) when no MFI reduction ≥50% nor MFI doubling; 62.4% (±6.9%) when MFI fluctuating; and 52.7% (±4.0%) when MFI doubling. Log-rank *p* < 0.001. dnDSA, *de novo* donor-specific antibodies; MFI, mean fluorescence intensity.

Specifically analyzing DQ-dnDSA (*n* = 363, 49.7% of total dnDSA), the proportion of DQ was significantly lower in the graft loss group (53.7 vs. 43.3%, *p* = 0.006). The number of DQ-dnDSA with MFI available at the first occurrence was 346 (Table 5). At first occurrence, most DQ dnDSA had MFI > 3,000 (74.9%). A ≥ 50% MFI reduction was observed in 31.2% (*n* = 108), and 7.2% (*n* = 25) became stable negative. In 84/108 (77.8%) cases, the MFI reduction occurred without treatment. The MFI evolution was associated with 5-year death-censored allograft survival (Figure 4).

**TABLE 5:** MFI values at first occurrence and MFI evolution of DQ-dnDSA analyzed by Luminex^®^.

Variables	All DQ dnDSA (*n* = 346)
**MFI at first occurrence of dnDSA**	
• 1: MFI < 500	• 0.6%
• 2: MFI 500–999	• 2.9%
• 3: 1,000–2,999	• 21.7%
• 4: 3,000–9,999	• 42.8%
• 5: >10,000	• 32.1%
MFI evolution of dnDSA after first occurrence^∧^	
• 1: MFI doubling	• 26.6%
• 2: MFI reduction ≥ 50%	• 31.2%
• 3: MFI fluctuating (MFI doubling and reduction ≥ 50% at some point)	• 5.5%
• 4: Other	• 28.3%
• 5: No MFI evolution available	• 8.4%
dnDSA becomes negative MFI < 500 at some point during evolution	12.3%
dnDSA becomes constant negative (MFI < 500) (Stable negative)*	7.2%

^∧^MFI evolution independent of biopsy-proven rejection and treatments. *Stable negative dnDSA defined as MFI < 500 in every sample after the first negative sample. MFI, mean fluorescence intensity; dnDSA, *de novo* donor-specific antibody.

**FIGURE 5:**
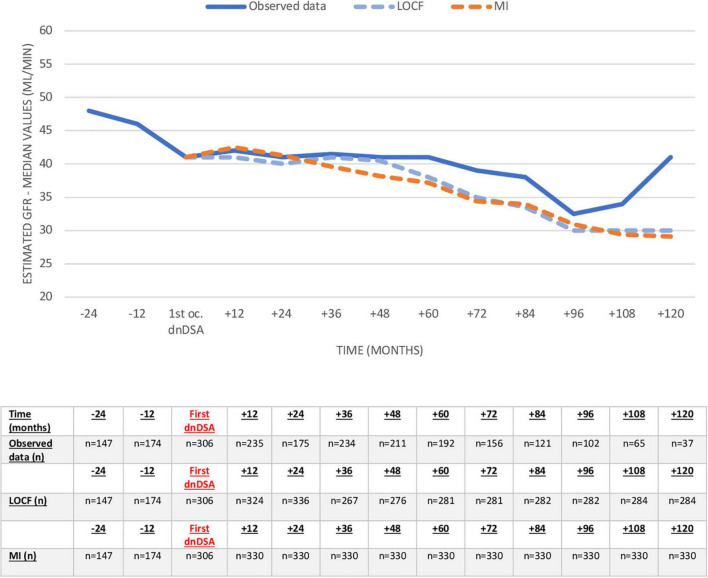
Evolution of eGFR (ml/min) before and after the first occurrence of the first dnDSA. Missing values after dnDSA were imputed using last observation carried forward (LOCF) analysis and multiple imputation (MI). eGFR, estimated glomerular filtration rate; dnDSA, *de novo* donor-specific antibodies.

**FIGURE 6:**
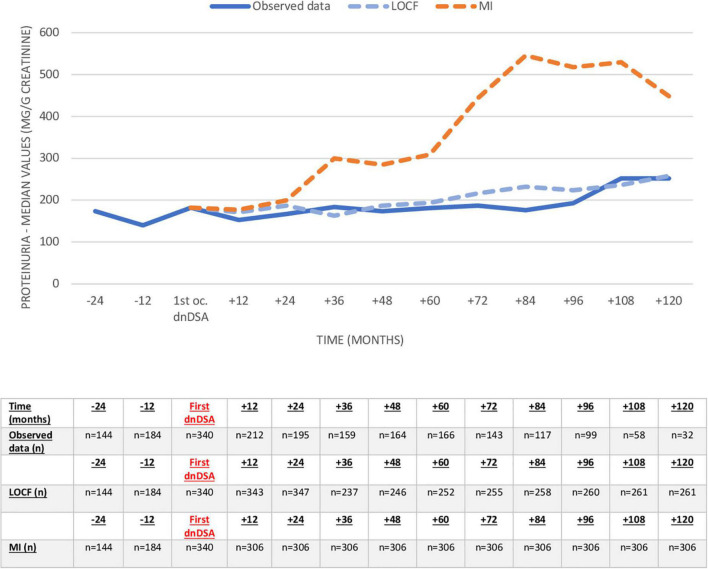
Evolution of proteinuria (mg/g creatinine) before and after first occurrence of first dnDSA. Missing values after dnDSA were imputed using last observation carried forward (LOCF) analysis and multiple imputation (MI). dnDSA, *de novo* donor-specific antibodies.

Proteinuria and eGFR (observed values, LOCF, and MI) before and after dnDSA appearance are shown in Figures 5, 6 and Supplementary Tables 1, 2. The eGFR was already decreased at the time of the first appearance of dnDSA, with a negative slope after this date (-11.9 ml/min/10 years), clearly demonstrating the importance of imputation compared to observed values. Conversely, proteinuria increased at the time of the first occurrence, and we observed increasing proteinuria over time, especially when we used the multiple imputation method.

Different patient characteristics were associated with death-censored graft loss in univariable Cox regression analyses (Table 6). Interestingly, patients with class II dnDSA had significantly less graft loss (*p* = 0.007), and the presence of both class I and II dnDSAs was significantly associated with graft failure (*p* < 0.001). Patients with ≥ 4 dnDSA experienced significantly more frequent graft loss (*p* < 0.001). DGF was associated with graft loss in univariable analysis, and conversely, those patients with a combined transplant experienced significantly less graft failure. Patients with doubling and fluctuating MFI values of dnDSA had significantly more graft loss (*p* < 0.001 and 0.008, respectively), while patients with ≥50% MFI reduction (*p* < 0.001) and stable negative MFI (*p* = 0.018) of dnDSA were significantly associated with less graft failure. These results were confirmed by multivariable Cox regression analysis (Table 7). MFI ≥ 50% reduction of dnDSA was associated with a positive outcome in the multivariable model; however, patients with doubling and fluctuating MFI values of dnDSA were not associated with graft loss. DGF was associated with graft failure in this model, and having at least one episode of TCMR or ABMR was an independent risk factor for graft loss. Other than expected, the class and number of dnDSA were not significant in multivariable analysis.

**TABLE 6:** Univariable Cox regression for death-censored graft loss.

Univariable Cox regression for death-censored graft loss	HR	CI 95% INF	CI 95% SUP	*p*
Patients with only class I dnDSA	0.7	0.4	1.2	0.310
Patients with only class II dnDSA	0.6	0.4	0.8	**0.007**
Patients with both class I and II dnDSA	2.1	1.5	3.1	**<0.001**
Patients with ≥ 2 dnDSA (independent of class)	1.4	0.9	1.9	0.053
Patients with ≥ 4 dnDSA (independent of class)	2.4	1.5	3.7	**<0.001**
Number of dnDSA per patient	1.2	1.0	1.3	**0.001**
MFI evolution of dnDSA*				
• Patients with MFI doubling of dnDSA (%) • Patients with MFI reduction ≥ 50% of dnDSA (%) • Patients with MFI fluctuating of dnDSA (MFI doubling and reduction ≥ 50% at some point) (%) • Patients with other MFI evolution of dnDSA (stable) (%)	1.9 0.4 1.8 1.3	1.3 0.3 1.1 0.9	2.7 0.7 2.8 1.9	< **0.001** < **0.001** **0.008** 0.114
Patients with stable negative MFI of all dnDSA**	0.3	0.1	0.8	**0.018**
Cold ischemia time (CIT, minutes)	1.0	1.0	1.0	0.106
Delayed graft function (DGF)	1.7	1.1	2.4	**0.004**
cPRA ≥ 5% at the time of TX (Eurotransplant)	1.1	0.7	1.8	0.433
cPRA ≥ 85% at the time of TX (Eurotransplant)	1.0	0.5	2.1	0.877
Donor type • Deceased donor • Living donor	1.1 0.9	0.7 0.6	1.5 1.3	0.593 0.593
First kidney transplant	0.8	0.5	1.3	0.486
Combined transplant	0.3	0.1	0.9	**0.040**
TCMR (all episodes, independent of first occurrence of dnDSA) (Banff 2017 Classification)	3.4	2.3	5.0	**<0.001**
ABMR (all episodes, independent of first occurrence of dnDSA) (Banff 2017 Classification)***	4.1	2.9	5.7	**<0.001**

*MFI evolution of at least one dnDSA of the patient. **Patients with all dnDSA stable negative (stable negative MFI defined as MFI < 500 in every sample after the first negative sample of dnDSA). ***All episodes of ABMR appeared at the time and/or after dnDSA first occurrence. dnDSA, *de novo* donor-specific antibody; MFI, mean fluorescence intensity; cPRA, calculated panel-reactive antibody; TX, transplant; TCMR, T-cell-mediated rejection; ABMR, antibody-mediated rejection. HR, hazard ratio; CI, confidence interval; SUP, superior; INF, inferior.

**TABLE 7:** Multivariable Cox regression analysis for death-censored graft loss.

Multivariable Cox regression for death-censored graft loss	HR	CI 95% INF	CI 95% SUP	*p*
Patients with only class II dnDSA	0.7	0.4	1.2	0.271
Patients with both class I and II dnDSA	1.2	0.6	2.3	0.467
Number of dnDSA per patient	1.0	0.9	1.2	0.512
MFI evolution of dnDSA*				
• Patients with MFI doubling of dnDSA (%) • Patients with MFI reduction ≥ 50% of dnDSA (%) • Patients with MFI fluctuating of dnDSA (MFI doubling and reduction ≥ 50% at some point) (%)	1.4 0.5 1.2	0.9 0.3 0.7	2.1 0.8 1.9	0.054 **0.012** 0.419
Delayed graft function (DGF)	2.0	1.3	2.9	**<0.001**
Combined transplant	0.9	0.3	2.5	0.874
TCMR (Banff 2017 Classification)**	2.5	1.7	3.8	**<0.001**
ABMR (Banff 2017 Classification)**	2.7	1.8	4.1	**<0.001**

*MFI evolution of at least one dnDSA of the patient. **All episodes, independent of the first occurrence of dnDSA. dnDSA, de novo donor-specific antibody; MFI, mean fluorescence intensity; DGF, delayed graft function; TCMR, T-cell-mediated rejection; ABMR, antibody-mediated rejection. HR, hazard ratio; CI, confidence interval; SUP, superior; INF, inferior.

## Discussion

It is well-known that dnDSA may appear years after transplantation and are strongly related to ABMR and graft failure (23–29). Despite a huge body of literature, little is known about the natural history of dnDSA and the clinical consequences beyond graft loss. In our study, we performed regular annual screening for HLA antibodies in a large and well-described population with 8 years of follow-up after dnDSA development. dnDSA developed only in 12% of the total cohort transplanted in this 21-year time period. The median time from transplant to the first appearance of dnDSA is around 3 years with a broad range. Graft failure occurred in 33.3% of patients, which is less than expected and probably related to regular dnDSA screening (30–32, 58), enabling early detection of dnDSA. Renal function was already deteriorated at the first occurrence, and in 6.5% of patients, rejection was present at that time. In total, 24.8% developed rejection over the follow-up period, which is clearly associated with poor results. Here, we describe fluctuating or increasing MFI values in a substantial number of patients, which is associated with inferior outcomes. In our cohort, 27.5% of patients have doubling MFI of dnDSA during follow-up. However, for the first time, we also describe a relevant cohort of patients (9.5%) with a stable disappearance of dnDSA, associated with better outcomes. In summary, our study provides detailed and granular clinical data for the natural history of dnDSA, which provides a solid basis for further studies and risk stratification.

Due to the strong association between the development of anti-HLA antibodies after transplantation and graft failure, sequential monitoring of HLA antibodies posttransplant has been recommended in different studies (16–18). Although there are clear recommendations for the screening of dnDSA when there is impaired kidney function, the universal screening and its frequency in stable patients is not well established (30–32). In our patients the median time to dnDSA positivity after the last negative test result is 11.3 months, supporting regular annual screening even in low risk, pretransplant DSA-negative patients. Almost half of the patients (43.5%) developed > 1 dnDSA, which was detected in most patients at first occurrence and in the others after a median of 14.4 months. These results support the value of annual screening for HLA antibodies after kidney transplantation for early detection of dnDSA.

Different risk factors for the development of dnDSA are described, with high HLA mismatch being one of the most important factors (33–36). As expected, a greater HLA-A and -B mismatch is significantly related to class I dnDSA formation in our cohort, and conversely, higher HLA-DRB1 and HLA-DQB1 mismatches are associated with class II dnDSA development. Thus, our study provides additional evidence for good HLA matching, which might be the easiest way to prevent the development of dnDSA. Graft inflammation, such as TCMR, can increase immunogenicity and can also precipitate the formation of dnDSA (23, 28). We can confirm this strong association, as around one-third of our patients had experienced TCMR before the appearance of dnDSA. Despite this, our study was not designed to specifically evaluate potential risk factors for the development of dnDSA in detail, since this was not the objective of our analysis.

The important role of dnDSA in the development of ABMR and graft dysfunction is well defined (23–26). ABMR was already present in 6.5% of patients at first occurrence and increased to 24.8% after around 8 years of follow-up. It has been described that class I dnDSA are more related to active ABMR, and conversely, class II dnDSA are commonly associated with chronic changes (23, 27, 37, 38), which is confirmed in our large cohort. As expected, the development of ABMR is significantly associated with a 2.7-fold higher risk of graft loss in multivariable analysis. Surprisingly, TCMR was also strongly associated with graft loss (HR 2.5), which might be explained by the local inflammation produced by TCMR, and tubulitis may result in subsequent irreversible nephron injury (59), which supports previous observations that TCMR is an independent and important risk factor for graft loss (5, 60, 61).

Previous literature suggested that class I dnDSA are less common and may appear sooner, while class II dnDSA, especially DQ, are frequently found and related to rejection and graft dysfunction (23, 27, 37–41, 62). In our study, there are no differences according to the class of dnDSA in the time of appearance after transplantation. We confirm that class II-DQ dnDSA are the most common dnDSA and potentially less harmful. In our analysis, the combination of class I and II dnDSA in particular has a negative impact on graft survival in univariable analysis, which, however, was not supported in the multivariable model, being in line with previous studies (27, 43). The impact of the number of dnDSA per patient, independent of class, on graft survival is not known yet, since it has not been specifically analyzed in previous studies. In our cohort, 43.5% of patients have >1 dnDSA, and a higher number of dnDSA per patient is associated with inferior 5-year graft survival, although this is not supported by multivariable analysis.

Today, Luminex^®^-based SAB technology is standard, and provides semi-quantitative information on the antibody level through the MFI value (44–46). One of the main problems is the lack of consensus on MFI positivity thresholds (32, 44, 46). There is no clear association between the MFI level and clinical outcomes (44–48). In our center, the general MFI cut-off to determine positivity is 1,000, and most of the dnDSA have MFI ≥ 1,000 at first occurrence. However, 13.6% of dnDSA present MFI below the cut-off level. In this latter group, we defined dnDSA by plausibility, epitope sharing, and other factors beyond the simple MFI value (52). We observed higher MFI in patients with class II dnDSA at the time of the first appearance. Interestingly, we did not observe a clear relationship between MFI values at first occurrence and outcome. Therefore, our data do not support a fixed MFI threshold, as low plausible MFI values also may have detrimental effects. Instead, our data provide further evidence for the complexity and limitations of MFI values and their interpretation.

It has been described that changes in MFI values <25%, or in some cases <50%, are not considered clinically important (32), but until now no studies have analyzed the MFI evolution of dnDSA and its relationship with graft failure in greater detail. The evolution of MFI in our study is analyzed by classifying dnDSA into three categories, with MFI reduction ≥50% being the most frequently observed category (36.9%). In these cases, only one-quarter had received specific active treatment for ABMR. As around 75% of patients with dnDSA did not develop clinical ABMR, our data suggest that the indication for allograft biopsy or potentially harmful treatment should be specifically evaluated in each case, since the appearance of dnDSA is not always associated with ABMR and a spontaneous reduction of MFI, and even stable negativity, without active treatment is frequent. Analyzing the relationship of MFI evolution with death-censored graft failure, MFI reduction ≥50% of dnDSA is a protective factor for graft loss, and this is supported by multivariable Cox regression analysis (*p* = 0.012). However, doubling and fluctuating MFI are related to graft failure in univariable analysis but are not contributors in the multivariable model (*p* = 0.054 and 0.419, respectively).

Specifically analyzing the MFI negativity, the proportion of dnDSA with stable negative MFI is 14.5%, with a greater MFI negativity in class I dnDSA (*p* < 0.001). Temporary and stable MFI negativity are significantly associated with better graft survival. This stable disappearance of dnDSA may have several causes, such as the development of anti-idiotypic antibodies that suppress DSA production (63, 64), which is not the objective of our analysis. Nevertheless, in our study, we show the relevance of stable negativity of dnDSA, this being related to better outcomes.

Evaluating DQ-dnDSA (39, 40, 62) MFI values and negativity, the MFI at the time of the first appearance is higher with MFI > 3,000 in 74.9%, and DQ-dnDSA are more persistent, being lower than the proportion of stable negative MFI (7.2%), although their presence is not significantly related to graft failure in our study. With our results, we can conclude that DQ-dnDSA are potentially less harmful to the graft or produce insidious and progressive chronic damage with late graft failure as described in some studies (23, 62); therefore, a longer follow-up is needed to evaluate long-term graft outcome. It has also been described that class II dnDSA, and therefore DQ, are usually non-complement binding IgG2 and IgG4 subclasses (23), suggesting a different, less studied, and complement-independent pathway of damage that could explain our findings. For the first time in our study, we provide important evidence about DQ evolution in our large cohort of patients, being the most frequent dnDSA after transplantation, presenting with higher MFI, and being more persistent. Accordingly, with our data, we support and highlight the need to expand knowledge about DQ-dnDSA and improve HLA-DQ matching strategies.

Changes in renal function were already registered in our study together with the first appearance of dnDSA. Although it has been described in some studies with sequential HLA antibody monitoring posttransplant that antibodies may appear before a rise in serum creatinine (17), in our cohort, renal function deteriorates at dnDSA first occurrence, and some patients already experience ABMR. Ten years after dnDSA, proteinuria and eGFR had worsened significantly, demonstrating the negative impact of dnDSA. Our data also highlight the importance of imputation methods, as results related to observed values are biased due to missing data in patients with graft loss.

The strength of our study is essentially to have a large and well-described cohort of patients with regular screening for DSA. In addition, our large and in-depth analysis of MFI by Luminex^®^ with long follow-up enables us to specifically evaluate the characteristics of the patients and the MFI evolution of each dnDSA. Furthermore, having allograft biopsies and analytical data available already at first dnDSA appearance makes it possible to correlate early clinical features with long-term clinical outcomes.

Nevertheless, our study has several limitations. This is a retrospective analysis, and we did not evaluate in depth a control group and did not analyze the factors that may be associated with dnDSA formation in greater detail. For such a study a different methodology (e.g., matched pairs and propensity score matching) is needed in order to avoid survival bias. In our cohort, adherence to treatment and levels of immunosuppressive drugs are not evaluated at the time of appearance of dnDSA. The analysis of dnDSA by Luminex^®^ with MFI data is currently the best tool available, although we must know the limitations of this assay. For patients with ≥50% reduction in MFI, we only registered specific active treatment for ABMR, but we were not able to analyze changes in chronic baseline immunosuppression. The evaluation of the class and level of dnDSA is key, but other characteristics, such as the complement binding capacity or IgG subclasses, also have an impact, which are not evaluated in our study as these tests are not performed routinely. Last classical antigen HLA mismatch is considered to describe dnDSA specificities in our cohort, without analyzing HLA epitope mismatch.

In summary, we are providing a large body of evidence for the natural course of dnDSA. We highlight the problem of the MFI positivity threshold, as even low, but plausible, MFI may have a negative impact. We confirm the high frequency of DQ dnDSA, presenting with higher MFI at the time of appearance and being more persistent, but seem less harmful to the graft. For the first time, we describe that MFI evolution is associated with graft survival, demonstrating the positive effect of a ≥50% reduction in MFI values, and we observed that almost 10% of patients became stable negative, which is related to better outcomes. Our large observational study provides important evidence for a better understanding of the evolution of dnDSA in renal allograft recipients. Further studies are needed to distinguish those dnDSA which are harmful from those dnDSA with an uneventful clinical course. A better knowledge of relevant HLA epitopes or the use of novel biomarkers of graft dysfunction, such as cell-free DNA, may provide additional information to identify patients at risk.

## Data availability statement

The original contributions presented in this study are included in the article/Supplementary material, further inquiries can be directed to the corresponding author/s.

## Ethics statement

Ethical review and approval was not required for the study on human participants in accordance with the local legislation and institutional requirements. Written informed consent for participation was not required for this study in accordance with the national legislation and the institutional requirements.

## Author contributions

CL, NL, and KB conceived and designed the study and wrote the article. MN and DSc provided technical support and acquired the data. CL analyzed and interpreted the data. NL, DSt, and SH performed HLA antibody testing. KW performed and interpreted histopathological examinations. MN, BO, AA, MC, FB, FH, and ES advised on the preparation of the article and provided conceptual advice. KB designed the study and supervised the research. All authors contributed to the article and approved the submitted version.

## Footnotes

1
https://www.etrl.org/vPRA.aspx
