# Supplementary material for: The natural history of de novo donor-specific HLA antibodies after kidney transplantation
Source: Front Med (Lausanne). 2022 Sep 16;9:943502. doi: 10.3389/fmed.2022.943502 (PMC9523126; doi:10.3389/fmed.2022.943502)
Supplement: Supplementary file 1 [file Data_Sheet_1.pdf]

Supplementary Material

Supplementary Figure 1. Survival analysis of death-censored graft failure in patients with dnDSA and without dnDSA ('Control group').

Patients with dnDSA of our study (n=400) had significantly lower graft survival after transplantation (Log rank $p<0.001$) compared to the 'Control group' *.

*'Control group': n=2752 patients without dnDSA (and without preformed DSA) in the same follow-up period of our study.

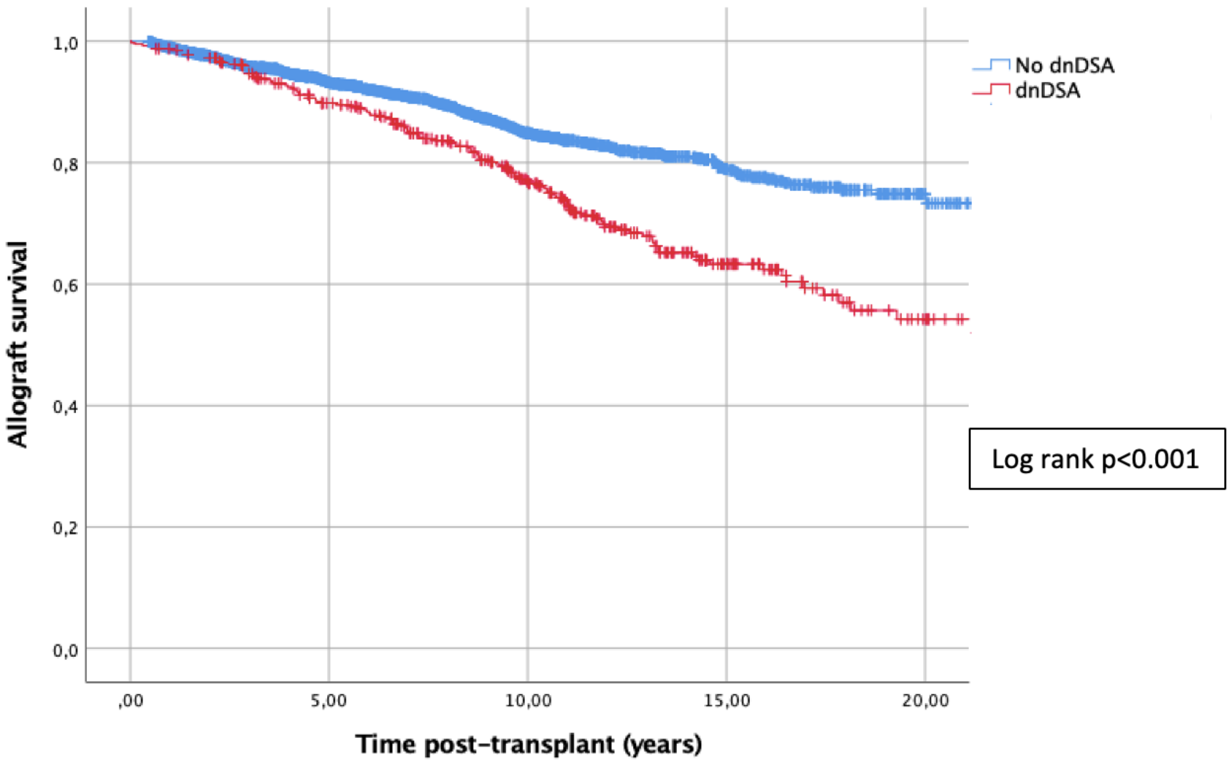

Supplementary Figure 2. Time to first occurrence of first dnDSA in 400 patients.

Median time from transplant to first occurrence of first dnDSA: 3.4 years (IQR: 1.3 - 7.3).

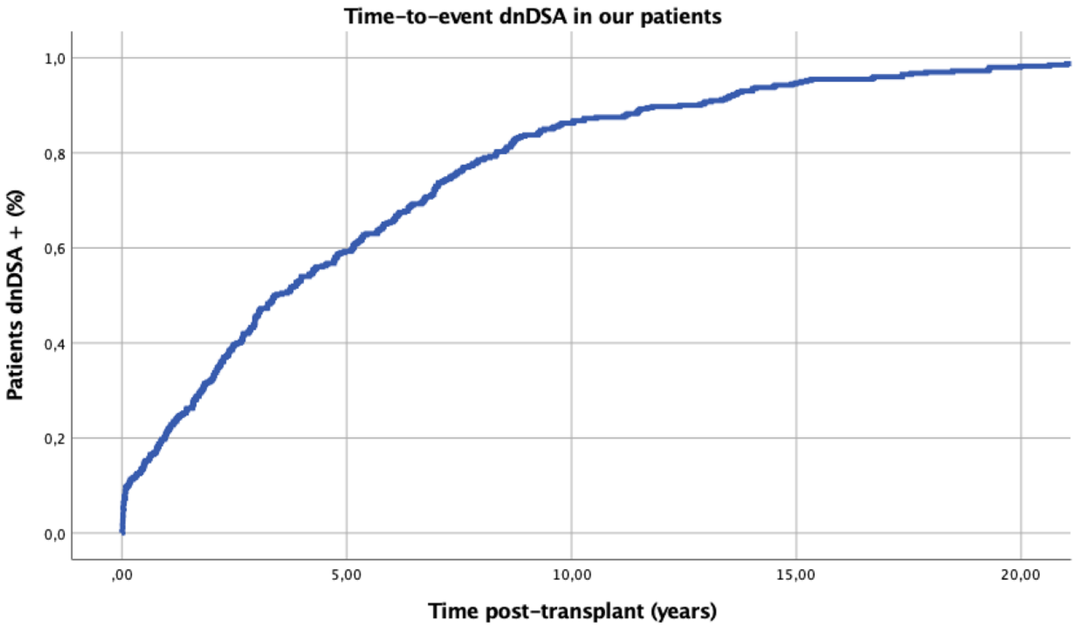

Supplementary Figure 3. Time to occurrence of another dnDSA (next dnDSA) after transplant in 174 patients with >1 dnDSA.

Median time from transplant to next dnDSA (≥1): 3.0 years (IQR: 1.3 - 7.1).

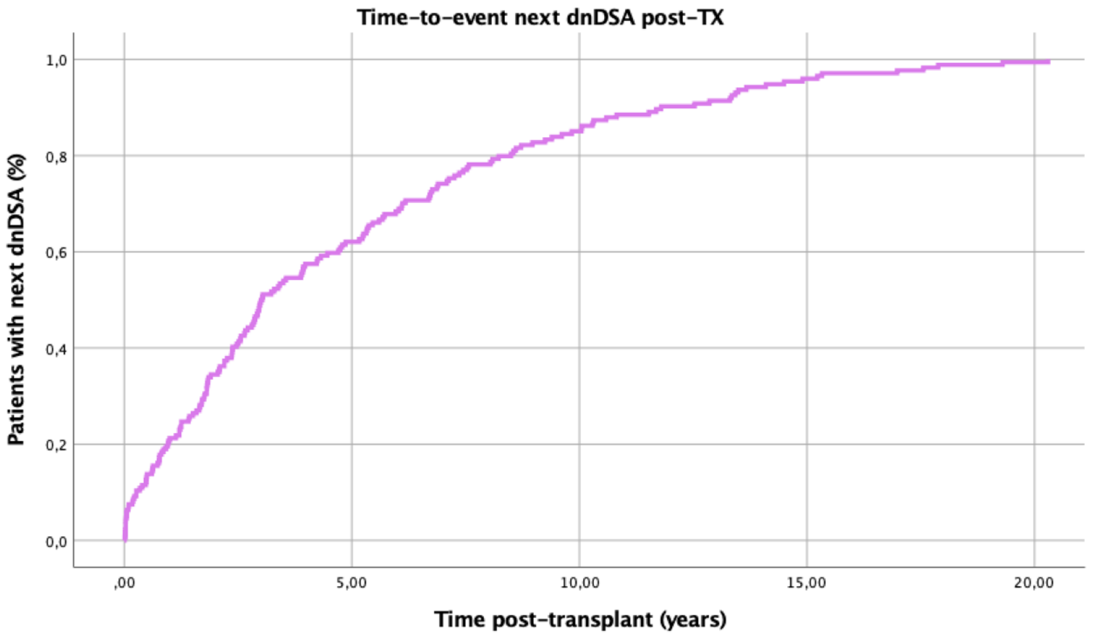

Supplementary Figure 4. Time to occurrence of another dnDSA (next dnDSA) from first occurrence of first dnDSA in 174 patients with >1 dnDSA.

Median time from first occurrence of first (≥1) dnDSA to next first appearance of another (≥1) dnDSA: 0.0 years (IQR 0.0 - 0.4).

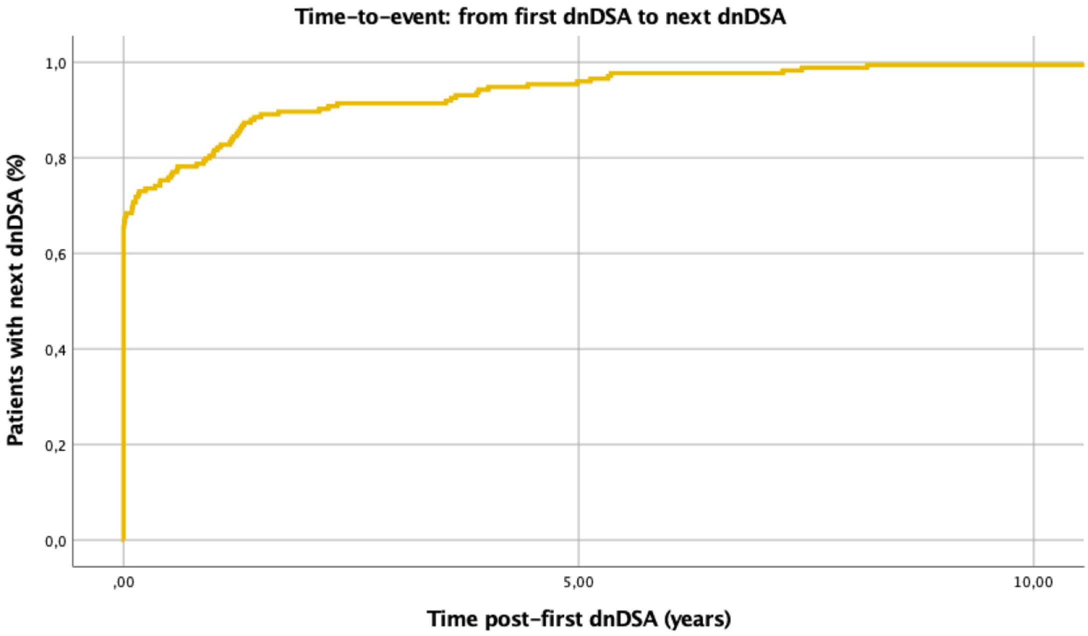

Supplementary Figure 5. Time to antibody-mediated rejection (ABMR) from first occurrence of first dnDSA in 99 patients.

Median time from first occurrence of first dnDSA to ABMR: 0.6 years (IQR: 0.0 - 2.5)

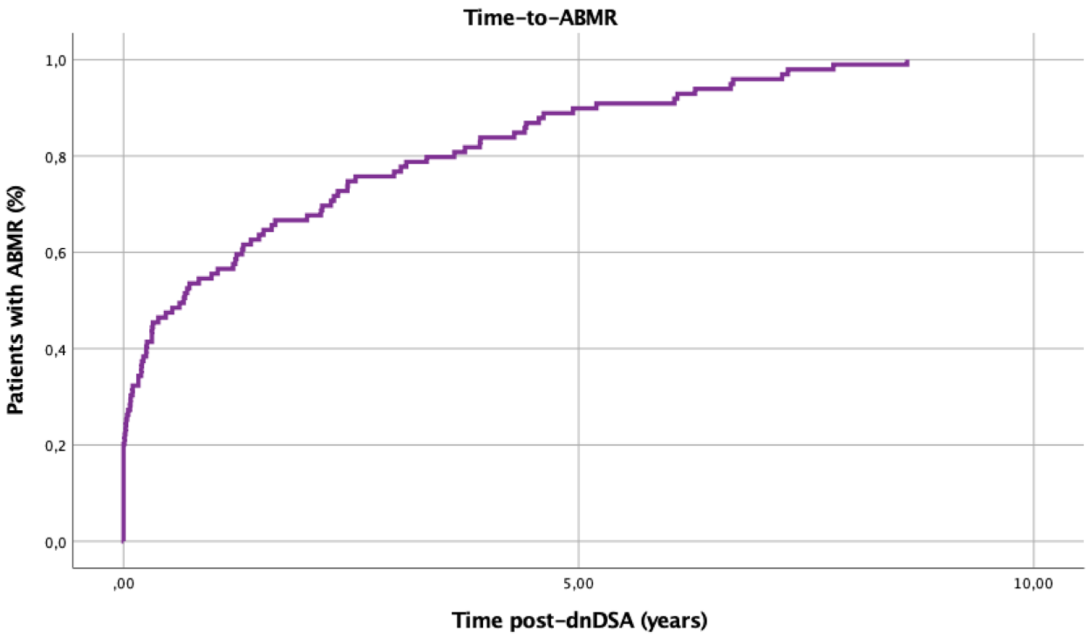

<u>Supplementary Figure 6. Survival analysis of death-censored graft failure and TCMR.</u>

1) Those patients with at least one episode of TCMR had significantly lower graft survival after transplantation (Log rank p <0.001).

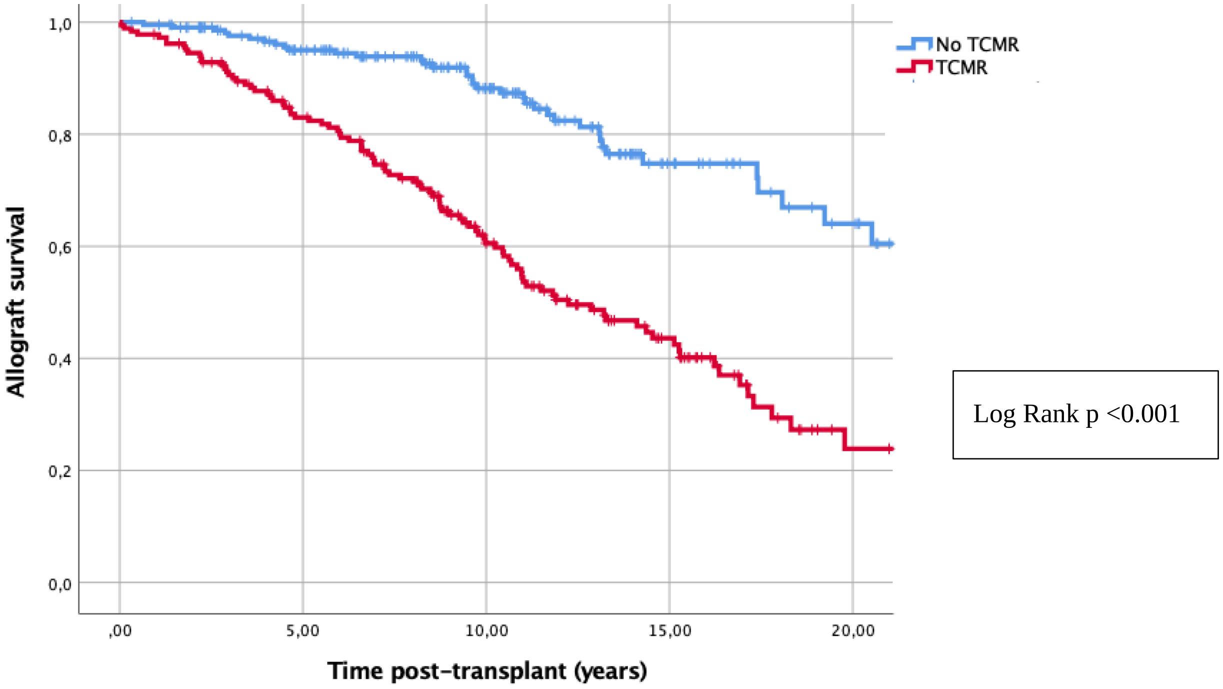

2) Those patients with at least one episode of TCMR had significantly lower graft survival after dnDSA appearance (Log rank p <0.001).

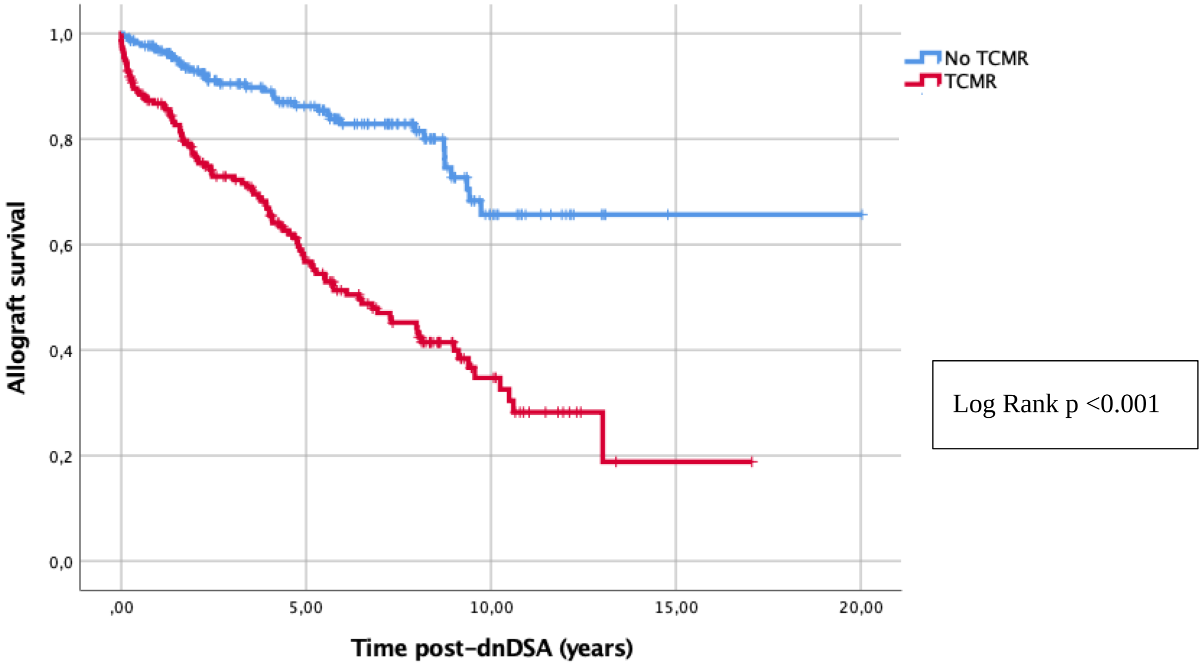

Supplementary Figure 7. Survival analysis of death-censored graft failure and ABMR

1) Those patients with at least one episode of ABMR had significantly lower graft survival after transplantation (Log rank $p < 0.001$).

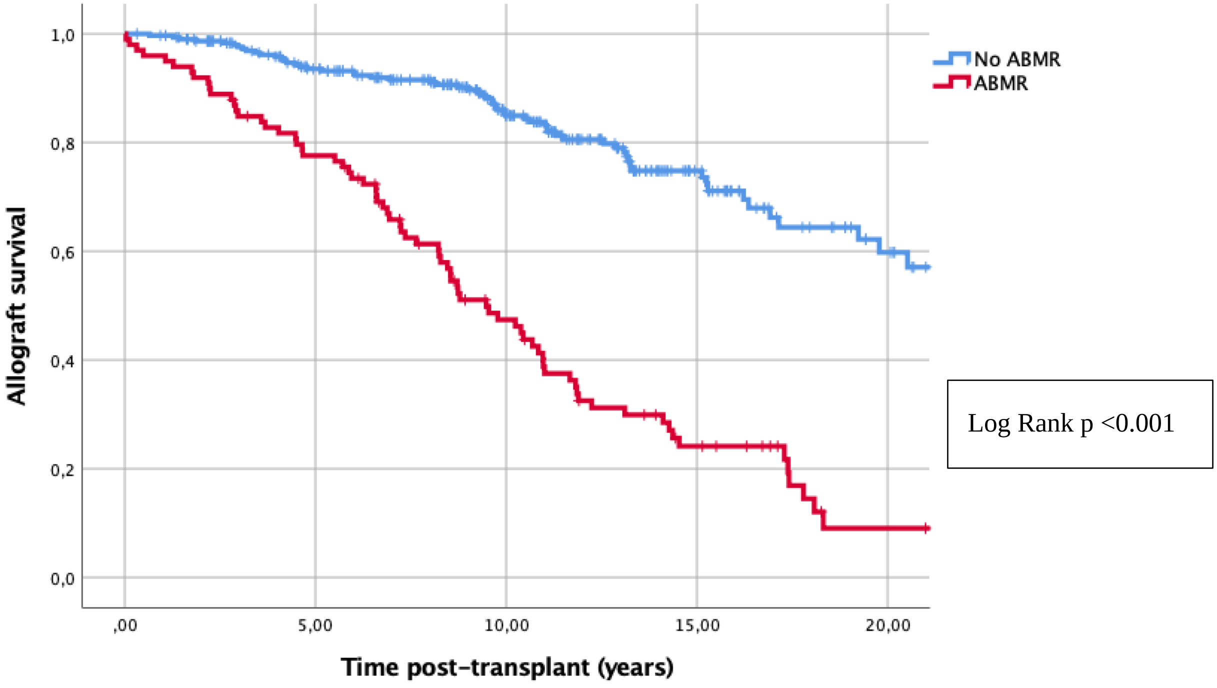

Log Rank $p < 0.001$

2) Those patients with at least one episode of ABMR had significantly lower graft survival after dnDSA appearance (Log rank $p < 0.001$).

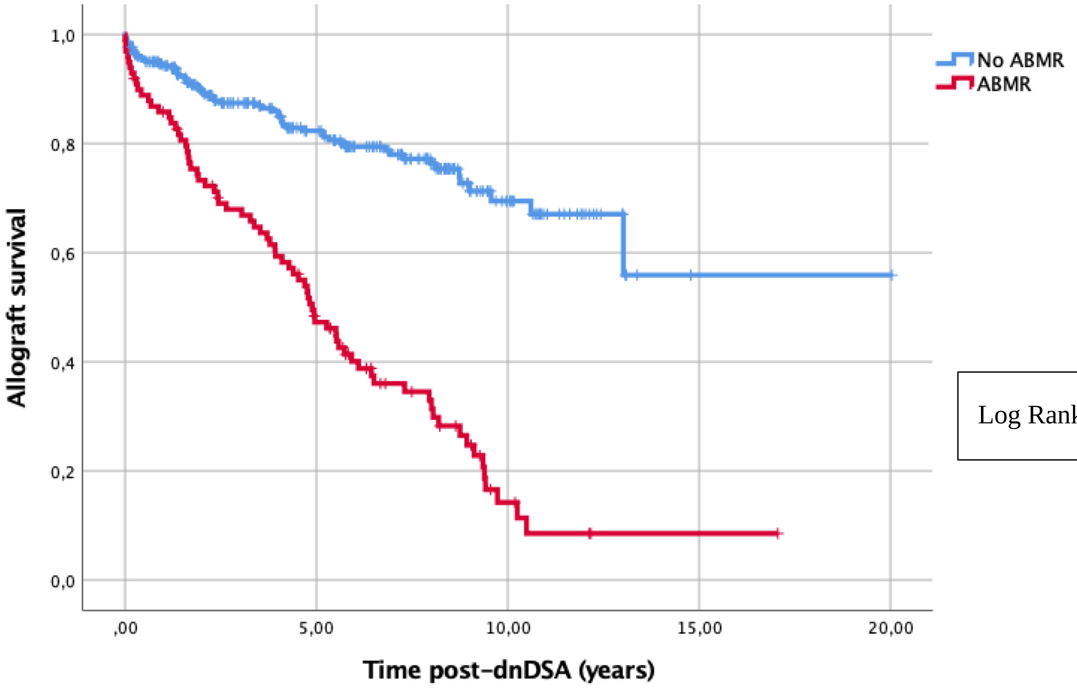

Log Rank $p < 0.001$

Supplementary Figure 8. Time to class I and class II dnDSA in 731 dnDSA.

No significant differences between time to class I and II dnDSA (log rank p=0.077).

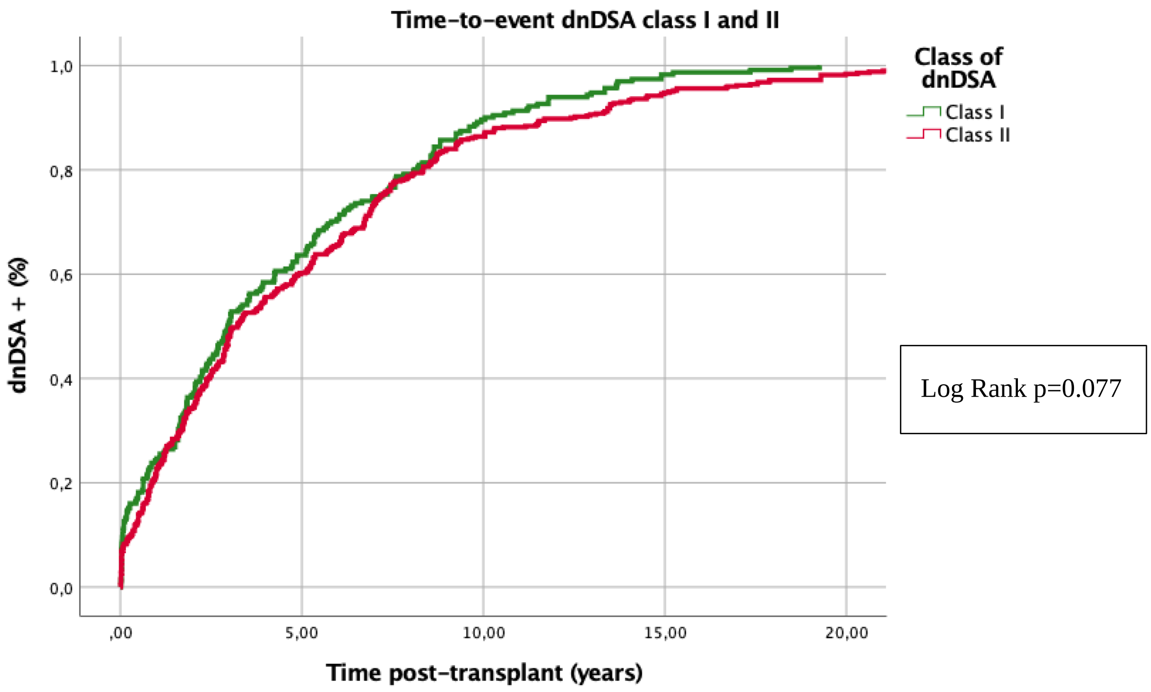

Supplementary Table 1. Observed data, last observation carried forward (LOCF) analysis and multiple imputation (MI) of estimated glomerular filtration rate - eGFR - values (ml/min) before and after first occurrence of first dnDSA.

Time (months)	-24mo (n=147)	-12mo (n=174)	First dnDSA (n=306)	+12mo (n=235)	+24mo (n=175)	+36mo (n=234)	+48mo (n=211)
Observed data (Median values, ml/min)	48.0	46.0	41.0	42.0	41.0	41.5	41.0
	-24mo (n=147)	-12mo (n=174)	First dnDSA (n=306)	+12mo (n=324)	+24mo (n=336)	+36mo (n=267)	+48mo (n=276)
LOCF (Median values, ml/min)	48.0	46.0	41.0	41.0	40.0	41.0	40.5
	-24mo (n=147)	-12mo (n=174)	First dnDSA (n=306)	+12mo (n=330)	+24mo (n=330)	+36mo (n=330)	+48mo (n=330)
MI (Median values, ml/min)	48.0	46.0	41.0	42.5	41.3	39.6	38.2

Time (months)	+60mo (n=192)	+72mo (n=156)	+84mo (n=121)	+96mo (n=102)	+108mo (n=65)	+120mo (n=37)
Observed data (Median values, ml/min)	41.0	39.0	38.0	32.5	34.0	41.0
	+60mo (n=281)	+72mo (n=281)	+84mo (n=282)	+96mo (n=282)	+108mo (n=284)	+120mo (n=284)
LOCF (Median values, ml/min)	38.0	35.0	33.5	30.0	30.0	30.0
	+60mo (n=330)	+72mo (n=330)	+84mo (n=330)	+96mo (n=330)	+108mo (n=330)	+120mo (n=330)
MI (Median values, ml/min)	37.2	34.4	34.0	30.9	29.4	29.1

Supplementary Table 2. Observed data, last observation carried forward (LOCF) analysis and multiple imputation (MI) of proteinuria values (mg/g creatinine) before and after first occurrence of first dnDSA.

Time (months)	-24mo (n=144)	-12mo (n=184)	First dnDSA (n=340)	+12mo (n=212)	+24mo (n=195)	+36mo (n=159)	+48mo (n=164)	+60mo (n=166)
Observed data (Median values, mg/g creatinine)	174.0	140.0	182.0	153.0	167.0	184.0	173.5	181.0
	-24mo (n=144)	-12mo (n=184)	First dnDSA (n=340)	+12mo (n=343)	+24mo (n=347)	+36mo (n=237)	+48mo (n=246)	+60mo (n=252)
LOCF (Median values, mg/g creatinine)	174.0	140.0	182.0	171.0	187.0	163.0	187.0	193.5
	-24mo (n=144)	-12mo (n=184)	First dnDSA (n=340)	+12mo (n=306)	+24mo (n=306)	+36mo (n=306)	+48mo (n=306)	+60mo (n=306)
MI (Median values, mg/g creatinine)	174.0	140.0	182.0	177.2	199.8	299.8	285.1	309.0

Time (months)	+72mo (n=143)	+84mo (n=117)	+96mo (n=99)	+108mo (n=58)	+120mo (n=32)
Observed data (Median values, mg/g creatinine)	187.0	176.0	193.0	252.0	252.0
	+72mo (n=255)	+84mo (n=258)	+96mo (n=260)	+108mo (n=261)	+120mo (n=261)
LOCF (Median values, mg/g creatinine)	216.0	232.5	223.5	236.0	258.0
	+72mo (n=306)	+84mo (n=306)	+96mo (n=306)	+108mo (n=306)	+120mo (n=306)
MI (Median values, mg/g creatinine)	443.7	545.4	517.9	529.7	448.3
